# Linac primary barrier transmission: Flattening filter free and field size dependence

**DOI:** 10.1002/acm2.13886

**Published:** 2023-01-04

**Authors:** Patrick N. McDermott, Douglas Drake, Cory Knill, Michael D. Sigler

**Affiliations:** ^1^ Beaumont Health System (now Corewell Health) Royal Oak Michigan USA; ^2^ Beaumont Health System Royal Oak Michigan USA; ^3^ Beaumont Health System Dearborn Michigan USA; ^4^ Beaumont Health System Lenox Michigan USA

**Keywords:** flattening filter free, primary barrier, shielding

## Abstract

There is widespread consensus in the literature that flattening filter free (FFF) beams have a lower primary barrier transmission than flattened beams. Measurements presented here, however, show that for energy compensated FFF beams, the barrier transmission can be as much as 70% higher than for flattened beams. The ratio of the FFF barrier transmission to the flattened beam barrier transmission increases with increasing barrier thickness. The use of published FFF TVL data for energy compensated FFF beams could lead to an order of magnitude underestimate of the air kerma rate. There are little data in the literature on the field size dependence of the barrier transmission for flattened beams. Barrier transmission depends on the field size at the barrier, not at isocenter Measurements are presented showing the relative dependence of barrier transmission on the field size, measured at the barrier, for 6 MV and 10 MV beams. An analytical fitting formula is provided for the field size dependence. For field sizes greater than about 150 cm in side length, the field size dependence is minimal. For field sizes less than about 100 cm, the transmission declines rapidly as the field size decreases.

## 1 INTRODUCTION

There is widespread consensus in the literature that flattening filter free (FFF) beams have the same or less primary barrier transmission compared to flattened beams.^1^, ^2^, ^3^, ^4^ Measurements reported here show that this is not true for Elekta energy compensated linac beams.

Official linac radiation shielding reports are: National Council on Radiation Protection and Measurements Report No. 151 (hereafter NCRP151), the Institute of Physics and Engineering in Medicine Report 75 (hereafter IPEM75) and the International Atomic Energy Agency Report 47 (hereafter IAEA47).^5^, ^6^, ^7^ There is no mention of FFF linac beams in NCRP151 as this document was published in 2005. IPEM75 implicitly assumes that the TVL values are the same for FFF as for flattened beams (IPEM75, page 5–7).^6^ In the textbook by Martin and McGinley it is stated that “There is an insignificant difference between the TVLs with and without the FFF mode at a given energy…^8^” The Elekta Oncology Products: Site Planning guide makes no distinction between the TVL of FFF versus flattened beams.^9^

The primary barrier transmission is defined here as:

(1) $$B=\left(\frac{{\dot{K}}_{a}}{{\dot{D}}_{0}\;S_{c}}\right)\left(\frac{d^{2}}{1\;\;m^{2}}\right)$$

where ${\dot{K}}_{a}$is the instantaneous air kerma rate measured in Sv/h ( = Gy/h) at a point that is 0.3 m beyond the distal face of the barrier, *d* is the distance from the source in meters, ${\dot{D}}_{0}$ is the instantaneous absorbed‐dose rate (workload in Gy/h) in a large water phantom on the central axis at a depth of *d_max_* and at a distance of 1.0 m from the source for a 10 × 10 cm^2^ field size and *S_c_* is the collimator scatter (head scatter) factor. If the beam calibration point is 1.0 m from the source at depth *d_max_* for a 10 × 10 cm^2^ field, and if 1 cGy = 1 MU at the calibration point, then ${\dot{D}}_{0}=\dot{\mu }\times 0.01\mathrm{Gy}/\mathrm{MU}$, where $\dot{\mu }$= the instantaneous “dose” rate (in MU/h). The MU/min can be read directly from the linac console.

For a flattening filter free (FFF) linac, there are two main considerations for barrier transmission: the beam energy spectrum and the beam profile. The presence of a flattening filter hardens the photon beam spectrum, making the average photon energy higher than for the beam incident on the filter. Removal of the filter, *by itself*, therefore results in beam softening. It is expected under these circumstances that the barrier transmission should decrease. If changes in the beam spectrum with field size and off‐axis distance are ignored, a FFF beam with a side length of 40 cm can be thought of as a superposition of a series of flat beams with different field sizes ranging from 0 to 40 cm. Under these circumstances, it would be expected that the beam transmission would be the weighted average over field size and thus smaller than that for a 40 cm flat field. A reasonable conclusion is that the barrier transmission for FFF beams should be lower than for flattened beams. Despite this, results reported here for 6 MV and 10 MV show that the air kerma/MU is higher for (energy compensated) Elekta FFF beams than for the corresponding flattened beam for every barrier measured.

For Varian linacs, the beam spectrum is softer in FFF mode. For 6 MV the percentage depth dose at a depth of 10 cm (10 × 10 cm^2^ field) is PDD(10) = 64.2% whereas for the flattened beam, PDD(10) = 66.4%. For 10 MV, PDD(10) = 71.7% for FFF and PDD(10) = 73.6% for the flattened beam. Elekta linacs, however, are energy compensated to keep the percentage depth dose at a depth of 10 cm unchanged. The PDD(10) is the same for 6 MV flattened and FFF beams (67.5%) and 10 MV flattened and FFF beams (73%). The off axis spectra, however, may vary between flattened and unflattened beams. Despite energy compensation, the FFF beam spectrum is not identical to the flattened beam spectrum. According to the Elekta Site Planning guide, *d_max_* for 6 MV FFF is 1.7 cm versus 1.5 cm for the flattened beam. For 10 MV, the FFF beam has *d_max_* = 2.4 cm compared to 2.1 cm for the flattened beam.^9^ Energy compensation is achieved by adjusting the accelerating potential.

Kry et al (2009) performed Monte Carlo calculations for 6, 10 and 18 MV (both FFF and flattened) using a Varian Clinac 2100 beam spectrum.^1^ These authors report an average reduction of 12% in TVLs (across all energies) and a decrease of 10%–20% in shielding thickness for 6 MV assuming all treatments are delivered in FFF mode.

Jank et al. report the results of their measurements for an Elekta linac that was custom modified.^2^ The TPR20/10 = 0.686 for both 6 MV FFF and flattened beams. For 10 MV, TPR20/10 = 0.735 for the flattened beam and TPR20/10 = 0.714 for the FFF beam. Measurements were made for 40 × 40 cm^2^ fields and normalized to the same dose rate. For primary barriers, these authors found that the ratio of the FFF transmission to the flattened transmission (*B*_FFF_/*B*_FF_) = 0.71 for 6 MV and 0.48 for 10 MV. Based on this, Xiao et al. conclude that: “For machines with energy‐restored FFF beams, there is still a shielding benefit, although it is slightly less. Primary barrier thickness was found to be up to 8% less.”^3^

Paynter et al. also report reduced penetration for 6 MV compensated FFF beams but not for 10 MV.^11^ These authors state that “For the 10 MV matched FFF the increase in instantaneous dose rate is greater than the proportional increased output of the machine.” They attribute this to the tuning of the beam requiring the maximum photon energy to be about 13 MeV. The maximum energy for a flattened 10 MV Varian beam is at most 11 MeV.^12^ The ratio ${\dot{K}}_{a}/\dot{\mu }$ for the energy matched FFF to the flattened 10 MV beam appears to be about 1.3. This is one of the few reported cases where the FFF beam is more penetrating than the flattened beam.

The data in Table 1 show that the quantity TPR20/10 is higher for FFF Elekta linac beams in comparison to FF beams.^13^ The data also show that Elekta FFF beams have a significantly higher TPR20/10 than the corresponding Varian FFF beams.

**TABLE 1:** TPR20/10 for FF and FFF beams^13^

Beam energy	Varian	Elekta
6 FF	0.666	0.678
6 FFF	0.630	0.684
10 FF	0.738	0.721
10 FFF	0.705	0.734

The field size dependence of barrier transmission is not discussed in NCRP151, IAEA47 or IPEM75. TVL values listed in NCRP151, IPEM75 and Martin & McGinley (except 10 MV) can be traced back to a paper published in 1984 by Nelson and LaRiviere.^14^ The field size used for these calculations is unclear, but is presumed to be large. The assumption of a 40 cm by 40 cm field size in official references for survey measurements is consistent with the ALARA principle, even though such large field size is rarely, if ever, used clinically. The relative field size dependence of barrier transmission for 6 MV and 10 MV flattened beams is reported here.

The purpose of this study is 2‐fold: (1) to examine the primary barrier transmission of energy compensated linac FFF beams in comparison to flattened beams with the same stated beam energy; and (2) to study the relative field size dependence of primary barrier transmission for flattened fields.

## 2 METHODS AND MATERIALS

Relative air kerma rate measurements are reported here for three Versa HD Elekta linac vaults for 6 MV (six barriers) and 10 MV (four barriers). Measurements were made on the central axis at a distance of 0.3 m from the outside surface of primary barriers. Readings were made using pressurized ion chamber survey meters all calibrated within the past year. The central axis was located by placing the ion chamber at the same height as the isocenter (1.3 m from the level floor) and by starting with a 3 × 3 cm^2^ field size to locate the lateral position of the central axis. The survey meters were not moved between measurements. Background radiation levels were 5–15 μR/h and these have been subtracted from the beam measurements. Data were collected for three different linac vaults designated L, RO and Db. The highest energy beam available in the “L” vault is 15 MV (flattened). For the “RO” vault the highest energy is 6 MV (both FFF and flattened) and for vault “Db,” the highest energy is 15 MV. For each beam the “dose rate” in MU/min was read from the console. The ion chamber readings were normalized by dividing by this number. For the relative field size dependence, the ion chamber readings have also been divided by *S_c_* for the relevant energy and field size. Each linac is calibrated so that the dose on the central axis at a depth of *d_max_* is 1.000 cGy/MU for a 10 × 10 cm^2^ field in a large water phantom at a distance of 100 cm + *d_max_* from the linac target. All FFF measurements were made with a field size of 40 × 40 cm^2^. In every case, the primary barriers were side walls. No ceiling measurements were made.

Based on the measurements, the ratio ${\dot{K}}_{a}/\dot{\mu }$ has been computed for both the FF and the FFF beams. It is assumed that this is equal to the ratio of the barrier transmissions (*B*_FFF_/*B*_FF_). Differences in *S_c_* between FFF and the flattened beam have been ignored for this portion of the study, but not for the field size dependence part of this study.

We have also measured the relative barrier transmission for flattened 6 MV and 10 MV beams for different field sizes at the barrier by varying the field size at isocenter. It is expected that *B* will depend on the size of the beam *at the barrier* (not at the isocenter) where the number of scattering centres is proportional to the beam area

## 3 RESULTS

The results of the measurements of air kerma rates for FFF versus flattened (FF) are listed in Table 2.

**TABLE 2:** Relative barrier transmission for FFF beams (Elekta ‐ Versa HD)

Barrier designation	Distance (m)^a^	Composition	Thickness (cm)	6 MV *B*_FFF_/*B*_FF_	10 MV *B*_FFF_/*B*_FF_
L1	8.1	concrete	183	1.53	1.14
L2	8.2	concrete	183	1.54	1.17
Db1	6.3	concrete/steel	160/25	1.77	1.18
Db2	6.9	concrete/steel	158/25	1.73	1.13
RO1	5.5	concrete/lead	91/18	1.08	——
RO2	5.5	concrete/lead	91/18	1.09	——

^a^ From target to distal barrier surface.

The data show that *B*_FFF_/*B*_FF_ for 6 MV beams is as high as 1.8 for the thickest barrier. For the 10 MV beams on the other hand *B*_FFF_/*B*_FF_ is never higher than 1.2. If we assume a single value of the TVL for each beam modality, the ratio of the barrier transmission for FFF to that for FF is given by:

(2) $$\frac{B_{FFF}}{B_{FF}}=10^{-t\left(\frac{1}{TVL_{FFF}}\;\;-\;\;\frac{1}{TVL_{FF}}\right)}$$

where *t* is the barrier thickness and TVL_FFF_ and TVL_FF_ are the TVL for the FFF and the FF beams, respectively. The ratio in Equation (2) depends on the thickness of the barrier as well as the difference in the TVL. Barriers L1, L2, Db1 and Db2 are much thicker than the barriers RO1 and RO2 because the L and Db barriers were designed to shield higher energy linacs. This may explain, at least in part, the high ratios for the 6 MV L and Db barriers.

It is possible to make a rough estimate of the TVL_e_ for the Elekta Versa HD 6 MV FFF beam based on the data in Table 2. We assume that $B_{FFF}≅10^{-t/TVL_{FFF}}$and that $B_{FF}≅10^{-t/TVL_{FF}}$. Taking the ratio of these two expressions for *B* and solving for TVL_FFF_ results in:

(3) $$TVL_{FFF}≅\frac{TVL_{FF}}{1-\frac{TVL_{FF}}{t}\log\left(\frac{B_{FFF}}{B_{FF}}\right)},$$

where *t* is the thickness of the barrier. If we assume that TVL_e_ = 33.0 cm (NCRP151) for a 6 MV flattened beam, then the data in Table 2, leads to TVL_FFF_ = 34.2 cm. Although this difference is relatively small, it can lead to a large difference in the value of *B*, especially for large values of *t*, as the TVL appears in an exponent. The FFF TVL_e_ listed in Table 1 of Kry et al. is 27 cm.^1^ In the worst case, using the Kry *et al*. TVL from a Varian 6 MV beam, and a concrete barrier of 180 cm thick, would lead to a difference of a factor of 20 in the transmission. The Kry *et al*. TVL data should not be used for Elekta linacs.

When the distance from the linac target to the barrier is sufficiently large, field divergence will cause the field to begin to impinge on the floor (or ceiling). In principle, under these circumstances, the effective field size on the barrier surface will be smaller than the actual field size. For a beam half‐opening angle of 14^o^ (assuming a collimator angle of 45^o^ and that isocenter is 1.3 m above floor level), this will occur when the proximal wall surface is > 5.2 m from the linac target. This will affect the linac barriers L1, L2, Db1 and Db2 in Table 2. The effect will be largest for barrier L2. For this barrier, the distance from the target to the proximal surface of the barrier is 6.4 m. The field area at the proximal barrier surface is 5.12 m^2^ and the area that is intercepted by the floor there is 0.09 m^2^. This is only about 2% of the total beam area at the proximal surface and is therefore negligible.

**FIGURE 1:**
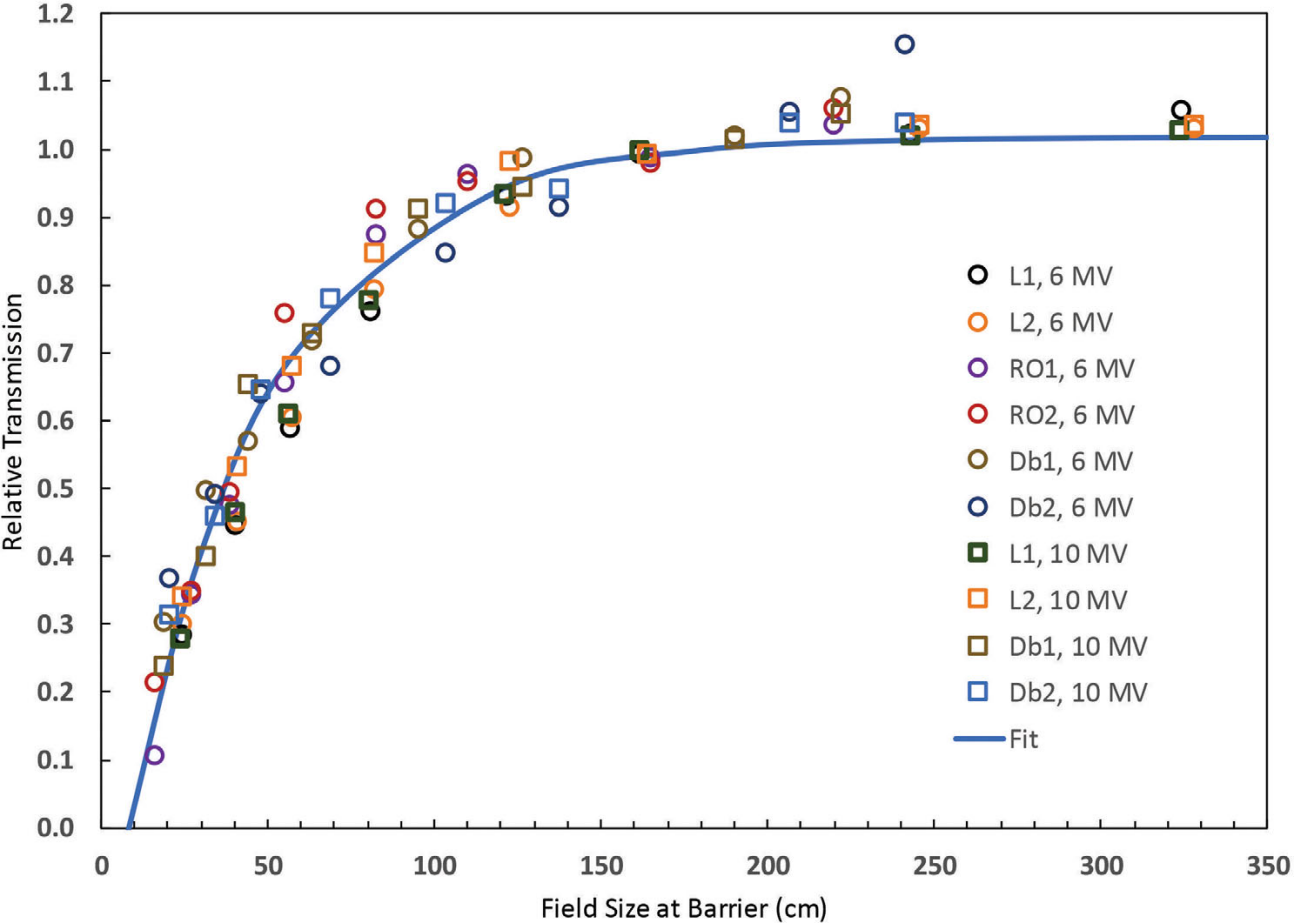
The relative field size dependence for primary barrier transmission for flattened 6 MV and 10 MV beams for six barriers with varying thickness. The side length of the square field is measured at the distal surface of the barrier. The relative field size dependence is normalized to 1.00 for field size of 180 cm. An analytic fit to this data is shown as the solid curve. The equation for this curve is given in the text

The results of the FF field size measurements are shown in Figure 1. This figure shows the relative transmission for different field sizes, normalized to 1.00 for a square field of side length 180 cm. The dispersion in the data for fixed field size may be due, in part, to differences in thickness of the barriers as well as the fact that data for 6 MV and 10 MV have been lumped together. The field size dependence may also depend on the barrier thickness. The field size dependence is insignificant for field sizes greater than about 150 cm. The relative transmission declines rapidly as the field sizes decreases below 100 cm. For a 10 × 10 cm^2^ field at isocenter and a distal barrier distance of 6 m, the relative transmission is about 70% of that for a 40 × 40 cm^2^ field (measured at isocenter). An analytic formula that provides a good fit to the data is given by:

(4) $$B\left(f\right)/B\left(180\right)=1.02\left(1-e^{-0.023(f-7.8)}\right)$$

where *f* is the side length of the square field (in cm, *f* > 10 cm) at the distal surface of the barrier. The fit is shown in Figure 1 as the solid curve.

## 4 DISCUSSION

Relatively small differences in TVL are amplified as the thickness of the barrier increases because the thickness appears in an exponent.

If the maximum beam energy in a linac vault does not have an FFF mode, the barrier will likely be significantly thicker than it needs to be for the lower energy FFF beam. In this case, the barrier transmission for the energy compensated FFF beam could be substantially higher than for the flattened beam with the same stated beam energy. Under these circumstances, the higher transmission of the FFF beam is likely to have an insignificant effect on barrier design unless the workload for this beam is excessive.

If the maximum beam energy for the linac does have a FFF mode, the increase in the transmission for an energy compensated FFF mode is likely to be on the order of 20% or less (for 10 MV). In this case, the effect of the FFF mode on shielding is likely to be minor provided that TVL_FF_ values are used and not the Kry et al TVL_FFF_ values.

## 5 CONCLUSION

The barrier transmission for energy compensated FFF beams can be greater than for the same energy flattened beam. This may occur if the maximum accelerating potential is higher for the FFF beam than for the flattened beam as it is for Elekta linacs.

For a linac whose highest energy has an energy compensated FFF mode, the barrier transmission may be slightly higher than for a flattened beam of the same stated energy and an extra margin on the order of 20% (beam transmission) is recommended. The TVL values listed in Table 1 of Kry et al. for FFF beams should not be used for Elekta linacs as this could result in air kerma rates about 20 times larger than expected. If the highest beam energy does not have an FFF mode and only lower beam energies have an FFF mode, then the increased transmission associated with the lower energy FFF mode is likely to be insignificant unless the workload for the FFF beam is especially high. In summary, the higher transmission of the FFF mode of energy compensated linacs is likely to have only a minor effect on shielding design provided that the FF values of TVL are used.

The relative field size dependence for six barriers and for 6 MV and 10 MV flattened beams has been measured. The field size dependence is described in terms of the field size at the distal surface of the barrier. There is little field size dependence for field size larger than about 150 cm (side length at the distal side of the barrier). For field sizes less than this the relative transmission declines as the field size decreases. A field with side length 60 cm (10 cm at isocenter, distance to distal barrier 6 m) has a transmission of about 70% of that for a 240 cm field (40 cm at isocenter). Equation (4) is a fitting formula for the relative field size dependence of the barrier transmission for 6 MV and 10 MV.

## AUTHOR CONTRIBUTIONS

McDermott suggested the idea for this study, analyzed the data and wrote the manuscript. Drake made measurements for one of the linacs and made suggestions and comments. Knill made measurements for one of the linacs and reviewed the manuscript. Sigler made measurements for one of the linacs and reviewed the manuscript.

## CONFLICT OF INTEREST

None.
